# Book Notices

**Published:** 1868-08

**Authors:** 


### Book Notices.

The Indigestions; or the Diseases of the Digestive Organs Functionally Treated. By THOMAS KING CHAMBERS, Honorary Physician to H.R.H., the Prince of Wales; Consulting Physician and Lecturer on the Practice of Medicine in St. Mary's Hospital; etc., etc., etc. Second American, from the Second and Revised London, Edition. Philadelphia: HENRY C. LEA. 1868. For sale by W. B. KEEN & Co., Chicago.

This volume contains nine chapters, on the following subjects: 1. Introduction; 2. Indigestion of various kinds; Habits of Social Life Leading to Indigestion; 4. Abdominal Pains; 5. Urinating; 6. Flatulence; 7. Diarrhœa; 8. Constipation and Costiveness; 9. Nervous Disorders connected with Indigestion. The writings of Dr. Chambers are too well known to need any commendation from us.

---

The Neuroses of the Skin: Their Pathology and Treatment. By HOWARD F. DAMON, A.M., M.D., Fellow of the Massachusetts Medical Society, etc., etc., etc. Philadelphia: J. B. LIPPINCOTT & Co. 1868. For sale by S. C. GRIGGS & Co., Chicago. Price $2.00.

This is an elegantly published monograph, of 114 pages; on a class of diseases obscure in pathology, and often obstinate in their continuance. Under the head of neuroses of the skin, the author treats of hyperæsthesia, dermatalgia, prurigo, urticaria, zoster, and anæsthesia. Several illustrative cases are given at the close of the volume. It will be found worthy of a careful perusal.

---

Materia Medica, for the Use of Students. By JOHN B. BIDDLE, M.D., Prof. of Materia Medica and General Therapeutics in Jefferson Medical College, etc., etc. Third Edition, with Illustrations. Philadelphia: LINDSAY & BLAKISTON. 1868. Pp. 384. Price $4.00. For sale by W. B. KEEN & Co., 148 Lake Street.

In this edition of his text-book on Materia Medica, the author has introduced quite a number of articles and topics omitted in the previous editions. Among them, are the several new anæsthetics, the sulphites and hyposulphites, the methods of hypodermic injections, and the atomizing of liquids. The work is increased both in size and value.

---

Circular No. 1. War Department, Surgeon-General's Office, Washington, June 10th, 1868. Report on Epidemic Cholera and Yellow Fever in the U.S. Army, during 1867.

This is a folio volume, or report, of 156 pages, containing observations and statistics of great value. on the subjects of cholera and yellow fever.

---

Lessons on Physical Diagnosis. By ALFRED L. LOOMIS, M.D., Prof. Institutes and Practice of Medicine in the Med. Dep't of University of New York, and Physician to Bellevue and Charity Hospitals. New York: ROB'T M. DEWITT Publisher, No. 11 Frankfort Street.

This is a well-published volume, of 159 pages. It is a brief, but excellent treatise on the methods of physical exploration in the diagnosis of diseases.

---

Atlas of Venereal Diseases, by A. CULLERIER, Surgeon to the Hospital Du Mida, etc., etc. Translated from the French, with notes and additions, by FREEMAN J. BUMSTEAD, M.D., Prof. of Venereal Diseases in the College of Physicians and Surgeons, N.Y., etc. Published by HENRY C. LEE, Phil.

The translation contains the text of Cullerier's work, with brief comments by Prof. Bumstead. Its chief merit, however, is in its illustrations, there being in the whole work twenty-six colored lithographic plates, containing about one hundred and fifty beautiful figures of venereal diseases. It is issued in five numbers, at three dollars each, of which three are already out.

As was above stated, the plates constitute the chief merit of the work, being extremely beautiful, and numerous enough to make a good gallery of venereal diseases. Equal praise cannot be given to the text. Cullerier is a loose reasoner, and only passably good in pathological description, while his discussion of the therapeutical branch of the subject is quite imperfect. Prof. Bumstead would have been the right man to correct these deficiencies, but he has limited his notes for the most part to a few brief comments on points wherein he differs from his author. This brevity was probably adhered to, in order to avoid increasing the bulk and expense of the work too much.

In his general method and style Cullerier shows good sense, but no precision or acuteness. In respect to the nature of syphilis he is a "unicist," that is, he admits only one virus for both the soft and the hard chancre, a position which may be considered as forever overthrown. The separate nature of soft and hard chancre, or, more properly speaking, of chancroid and true syphilis, is so intimately connected with the pathology and therapeutics of venereal diseases that one can by no means be a thoroughly reliable practitioner who does not understand the distinction between them.

In respect to gonorrhœa Cullivier commits the error of saying that the chronic form is not contagious, a most false and dangerous position to assume. The pathological connection between chronic gonorrhœa and granular urethritis, granular ophthalmia, and granular inflammation of the cervix uteri, has escaped his attention altogether. With characteristic French morality, he recommends those afflicted with chronic gonorrhœa to practice sextual intercourse, lest the erections caused by total abstinence from women should aggravate the inflammation.

Bumstead's notes have done much to correct the author's errors, but are not sufficient to rectify everything. As was remarked above, the merit of the work does not consist in its text, but in its superb colored plates, which, in fulness, beauty, and variety surpass anything previously published.

---

Klinik der Ohrenkrankheiten. Ein handbuch für Studirende und Aertze. Von Dr. S. Moos, Praktischer Artz und Docent au der Universität in Heidelberg. Wein. 1866. Wilhelm Brawmüller.

We have received from the publishers, through B. Westerman & Co., 440 Broadway, N. Y., this valuable hand-book of Dr. Moos upon diseases of the ear. The general, or first part, is devoted to the physical examination of the organs of hearing, embracing the outer, middle, and inner ear, and including a description of the various instruments made use of for this purpose. The value of Phinoscopy in the diagnosis of the diseases of the middle ear is fully recognized, and directions for the manipulation, taken chiefly from Semeleden, are given in detail.

In the chapter devoted to the examination of the inner ear, the theory of Helmholtz is discussed, and its value considered. The second, or special part, is devoted to the diseases of the ear. In the first six chapters, the author discusses the affections of the ear—embracing the inflammations, acute and chronic, circumscribed, diffused, of this portion of the organ. In the next eight chapters are considered the pathological changes in the tympanum, and the diseases of the middle ear. The remainder of the volume is devoted to the diseases of the nervous apparatus.

From the somewhat hasty examination which we have given to the work, we have no hesitation in recommending it to those of our readers who are interested in German medical literature. To the specialist in this department no recommendation of ours will be necessary.

